# Efficacy and safety of topical clobetasol propionate in comparison with alternative treatments in oral lichen planus: an updated systematic review and meta-analysis

**DOI:** 10.3389/fmed.2024.1391754

**Published:** 2024-05-24

**Authors:** Tao Zheng, ChengYong Liu, YeTong Wang, Rong Zhou, Dan Wu, Jin Tan, KeKe Zhu

**Affiliations:** ^1^School of Stomatology, Hunan University of Chinese Medicine, Changsha, Hunan, China; ^2^Changsha Hospital for Maternal and Child Health Care, Changsha, Hunan, China; ^3^Department of Stomatology, The First Affiliated Hospital of Hunan University of Chinese Medicine, Changsha, Hunan, China

**Keywords:** oral lichen planus, systematic review, meta-analysis, clobetasol propionate, corticosteroids

## Abstract

**Background:**

Oral lichen planus (OLP) is a relatively common chronic T cell-mediated disease characterized by pain and inflammation. Clobetasol propionate (CLO) is the first-line drug in the treatment of OLP. The meta-analysis aimed to evaluate the efficacy and safety of CLO for treating patients with OLP.

**Methods:**

PubMed, Embase and Web of Science were systematically searched from the database inception date up to August 2023. There were no restrictions on language or date of publication. The outcomes of our interest were as follows: improvement of clinical signs and/or symptoms, total lesion size, relapse and adverse events.

**Results:**

A total of 17 RCTs evaluating the effects of CLO were included in this study. The results revealed no significant difference in the clinical score (WMD = 0.14, 95% CI: −0.39, 0.66; *p* = 0.609) and pain score (WMD = 0.17, 95% CI: −0.44, 0.79; *p* = 0.582) between CLO and other treatments. However, clinical resolution (RR = 1.61, 95% CI: 1.17, 2.22; *p* = 0.003) and symptoms improvement (RR = 1.80, 95% CI: 1.17, 2.77; *p* = 0.008) were significantly different between CLO and other treatments. Moreover, there was a significant reduction in the total lesion size with CLO treatment (WMD = -0.58, 95% CI: −1.03, −0.13; *p* = 0.011). In addition, CLO showed no statistical incidence of adverse events (RR = 1.46, 95% CI: 0.86, 2.50; *p* = 0.161) and relapse (RR = 1.56, 95% CI: 0.66, 3.71; *p* = 0.314) than other therapies.

**Conclusion:**

This systematic review and meta-analysis of 17 randomized clinical trials supported the long-term application of CLO as an effective regimen in OLP patients.

## Introduction

Oral lichen planus (OLP) is a common T-cell-mediated autoimmune inflammatory (1). The global prevalence of OLP is 1–2%, typically appearing in the fourth decade of life, and more commonly affects women than men (2–4). The feature of OLP lesions in the buccal mucosa are normally white reticular, which are frequently asymptomatic (5). Conversely, the symptoms of ulcerative or erosive lesions vary from mild discomfort to severe pain that may negatively affect quality of patient’s life (6, 7). OLP is clinically unpredictable, with worsening over time (8). In fact, due to the cases of relapse are not uncommon, long-term symptoms relief is a great challenge in treating OLP (9). More importantly, an analysis of the existing literature suggests that patients diagnosed with OLP possess an increased risk of developing oral squamous cell carcinoma (OSCC) (10, 11). Consequently, OLP has been classified as a potentially malignant oral disorder (12).

While significant strides have been made in OLP research, a definitive cure and effective treatment for OLP is yet to be discovered (13). Most therapeutic strategies, given the present scenario, are focused on relieving symptomatic pain and inflammation. In the management of non-erosive OLP, the use of topical corticosteroids, such as 0.05% clobetasol propionate (CLO), is commonly employed. For erosive OLP, localized treatment via triamcinolone acetonide injections is often utilized (14). However, in cases of severe erosive or refractory OLP, alternative therapeutic approaches have been explored. Systemic therapies such as systemic corticosteroids, apremilast, hydroxychloroquine, and systemic retinoids have been proposed for their immunomodulatory effects (3). Additionally, biologics targeting specific inflammatory pathways, such as anti-IL-13, anti-IL-12/IL-23, and anti-IL-23 monoclonal antibodies, have shown promising results in refractory cases of erosive OLP, suggesting a potential role in personalized treatment strategies (14, 15). Emerging non-pharmacological treatments, including CO2 laser therapy and photodynamic therapy, have also demonstrated varying degrees of efficacy, offering alternative options for patients with contraindications or poor responsiveness to conventional therapies (16, 17). Other novel therapies, such as low-level laser therapy (LLLT) and ozone therapy, have shown promising results in reducing pain and inflammation in OLP patients (18). However, it is important to note that their effectiveness may vary among different patients.

As it stands, CLO, a corticosteroid, is considered an effective anti-inflammatory and pain relief for managing mild symptoms of OLP (14). Alternative therapies are only recommended in some cases, such as those characterized by poor responsiveness, intolerance, or existing contraindications to corticosteroids (19). Recent studies have also indicated that the topical application of CLO demonstrated efficacy in treating erosive, refractory, or recurrent forms of OLP (20, 21).

To elucidate the clinical application scenarios of CLO in OLP treatment, this study conducted a comprehensive comparison of the efficacy and safety between CLO therapy and alternative treatments. Although current meta-analyses do not robustly support the superiority of CLO in treating OLP, the increasing number of randomized controlled trials (RCTs) centered on CLO has prompted an update of our review. This update aims to provide a more accurate assessment of the effectiveness and safety of CLO in treating OLP, thereby offering a more informed basis for clinical decision-making.

## Materials and methods

This meta-analysis adhered to the Preferred Reporting Items for Systematic Reviews and Meta-Analysis (PRISMA) and the revised intervention assessment methodology from the Cochrane Handbook (22, 23).

### Search strategy

We systematically searched PubMed, EMBASE and Web of Science, encompassing all relevant studies ranging from the database inception date up to August 2023. References of the selected articles were also manually searched to identify additional relevant studies. To avoid missing pertinent literature as much as possible, there were no restrictions on language, publication date, or type. Literature management was done using EndNote (X9), and two reviewers independently assessed all research. Discrepancies were negotiated to reach a consensus.

### Inclusion and exclusion criteria

The following inclusion criteria were employed: (1) RCTs involved patients diagnosed with OLP either clinically or through histopathology. (2) Original studies investigating the application of CLO in the treatment of OLP. (3) Placebo or alternative treatment as the control. (4) At least one outcome regarding efficacy, safety, and stability. We excluded the following studies: (1) Reviews, case reports, *in vitro* research, letters, book chapters, and conference papers. (2) Studies with insufficient data and inability to establish contact with authors.

The primary efficacy outcomes comprised clinical response (score and resolution) and symptoms response (pain score and symptoms improvement). Clinical response was evaluated through the Thongprasom scale and signs improved, categorized into complete resolution and partial or no resolution. The secondary outcomes incorporated total lesion size, relapse rate post-treatment, and safety as determined by the proportion of patients with adverse reactions at any study phase.

### Data extraction and management

Two investigators (Z.T. and W.Y.T) independently screened articles for relevance. Essential data from full texts, including the first author’s name, publication year, geographical location, sample size, interventions, and outcomes, were extracted. Any differences between reviewers were consulted by the third reviewer (L.C.Y) to ensure consistency.

### Quality assessment

The risk of bias of all the included RCTs was evaluated by two independent investigators (Z.T. and W.Y.T.) with Cochrane ROB_2 (24). Disagreements were resolved by discussions with the third reviewer (L.C.Y) until a consensus was reached. These trials were examined across seven different RCT domains (25), including random sequence generation (selection bias), allocation concealment (selection bias), the blinding of participants and personnel (performance bias), blinding of outcome assessment (detection bias), incomplete outcome data (attrition bias), selective reporting (reporting bias), and other sources of bias. For each domain, the risk of bias was assessed as low, high, or unclear based on the Cochrane Collaboration guidelines (25). If all domains showcased low risk, then the study was deemed to have an overall low risk of bias. If the study was high risk in any one domain, then it was classified as having an overall high risk of bias. Otherwise, the study was considered to be unclear risk.

### Statistical analysis

We employed the Stata 12.0 software to quantify treatment effects. For dichotomous data, risk ratios (RR) with 95% confidence intervals (CIs) were calculated, while continuous outcomes were represented by weighted mean differences (WMD) with 95% CIs. Heterogeneity among the included studies was assessed using the Q and I^2^statistics, in which *p* < 0.1 or I^2^ > 50% indicated significance. In the presence of significant heterogeneity, a random-effects model was adopted (26); otherwise, a fixed-effect model was chosen (27). For potential publication bias evaluation, Begg (28) and Egger’s (29) tests were applied when 10 or more studies were included in the analysis. Subgroup analyses were stratified by intervention treatment, control treatment and treatment duration. A sensitivity analysis was conducted by excluding one study at a time. All statistical tests were two-sided, with *p* < 0.05 being indicative of statistical significance.

## Results

### Literature selection and included studies

**Figure 1:**
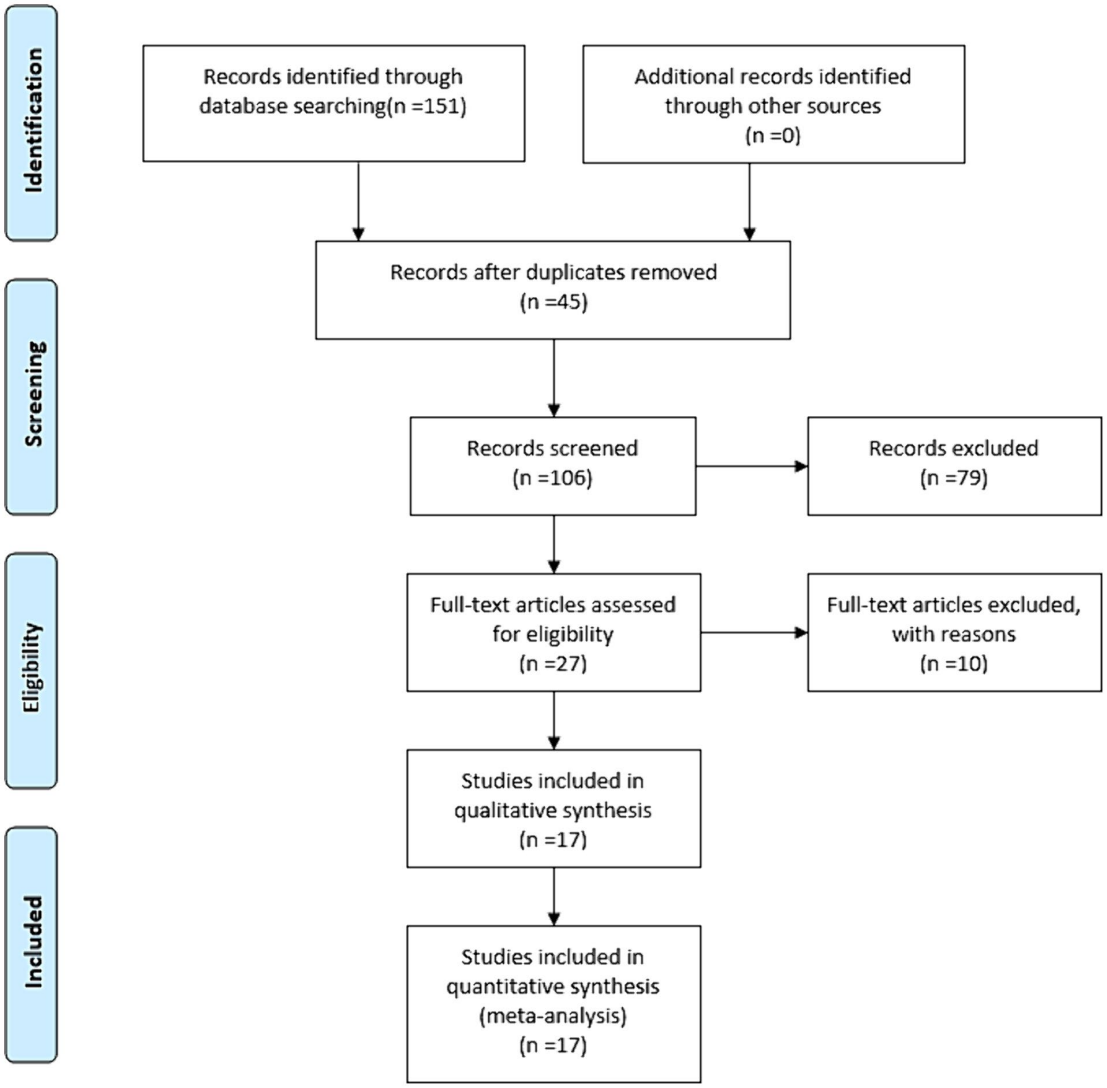
Eligibility of studies for inclusion in meta-analysis.

The flow diagram of the literature search and study selection process is depicted in Figure 1. Based on our established search strategy, a total of 151 relevant studies were identified. From these, 45 were excluded due to duplication, and an additional 79 were eliminated after evaluating their titles and abstracts. This left 27 articles for a full-text review. Following this review, 17 studies were chosen for the meta-analysis. The details and characteristics of these studies are summarized in Table 1.

**Table 1:** Baseline characteristics of patients in the trials included in the meta-analysis.

Study	Country	Age, years, mean ± SD or mean age (range)	Intervention drug	Control drug	Sample size, *n*	Female, %	Duration
Mamadapur 2022 (30)	India	42.57 (CLO) 44.63 (coconut)	CLO ointment 0.05% (twice daily)	Coconut cream 50% (twice daily)	30/30	70	4 weeks
Brennan 2022 (31)	USA	58.6 ± 11.8 (20 μg CLO)	CLO patch 20/5/1 ug (twice daily)	PBO (twice daily)	33/34/40/31	72	4 weeks
		59.7 ± 10.5 (5 μg CLO)					
		62.2 ± 12.1 (1 μg CLO)					
		63.9 ± 11.5 (PBO)					
Kumar 2022 (32)	India	48.7 ± 13.3 (CLO)	CLO ointment 0.05% (twice daily)	N. sativa cream 75% (twice daily)	30/30	62	6 weeks
		44.5 ± 13.5(N. sativa)					
Ferri 2021 (33)	Brazil	62.2(30–83)	CLO ointment 0.05% (3 times daily)	PDT (twice a week)	17/17	94	17 weeks
Santonocito 2021 (34)	Italy	65.55 ± 9.61 (CLO)	CLO ointment 0.05% (twice daily)	AIM (3 times daily)	18/20	47	12 weeks
		62.5 ± 9.13 (AIM)					
Arduino 2018 (35)	Italy	70(52–81) (CLO)	CLO cream 0.05% (twice daily)	PBO (twice daily)	18/18	83	8 weeks
		66(50–79) (PBO)					
Hettiarachchi 2017 (36)	Sri Lanka	46.88 ± 12.13 (CLO)	CLO ointment 0.05% (twice daily)	TAC cream 0.1% (twice daily)	34/34	63	5 weeks
		46.65 ± 13.15 (TAC)					
Sivaraman 2016 (37)	India	39.77 (18–65)	CLO ointment 0.05% (4 times daily)	TAC ointment 0.03% (4 times daily)	10/10/10	60	6 weeks
Dillenburg 2014 (38)	Brazil	61.33 ± 11.85 (CLO)	CLO ointment 0.05% (3 times daily)	PDT (three times a week)	21/21	83	12 weeks
		55.14 ± 15.96 (PDT)					
Sonthalia 2012 (39)	India	34.35 ± 16.20(CLO)	CLO ointment 0.05% (twice daily)	TAC ointment 0.1% (twice daily)	20/20	60	8 weeks
		35.05 ± 13.24(TAC)					
Cilurzo 2010 (40)	Italy	51.5 (32–72)	CLO tablets 24 ug (3 times daily)	PBO (3 times daily)	16/16/16	54	4 weeks
			CLO semisolid 123 ug (3 times daily)				
Corrocher 2008 (41)	Italy	43.7 ± 18.2 (CLO)	CLO ointment 0.05% (4 times daily)	TAC ointment 0.1% (4 times daily)	16/16	63	6 weeks
		43.6 ± 19.3 (TAC)					
Radfar 2008 (42)	USA	58(36–78) (CLO)	CLO ointment 0.05% (4 times daily, dosage decreased gradually)	TAC ointment 0.1% (4 times daily, dosage decreased gradually)	14/15	55	6 weeks
		59(29–77) (TAC)					
Conrotto 2006 (43)	Italy	67.95 ± 7.91 (CLO)	CLO ointment 0.025% (4 times daily)	CYC 1.5% (4 times daily)	19/20	64	8 weeks
		63.4 ± 9.59 (CYC)					
Carbone 1999 (20)	UK	61.2 ± 10.74 (CLO)	CLO ointment 0.05% (twice daily, dosage decreased gradually)	FLU 0.05% (3 times daily, dosage decreased gradually)	25/24/11	63	26 weeks
		60 ± 9.75(FLU)		PBO (3 times daily, dosage decreased gradually)			
		62 ± 11.22 (PBO)					
Sardella 1998 (44)	Italy	61(34–84)	CLO ointment 0.05% (twice daily)	MES 5% (twice daily)	14/11	64	4 weeks
Rodstrom 1994 (45)	Sweden	58(41–77)	CLO ointment 0.05% (twice daily, dosage decreased gradually)	TRI acetonide 0.1% (twice daily, dosage decreased gradually)	20/20	75	9 weeks

CLO, clobetasol; PBO, placebo; N. sativa, Nigella sativa; AIM, Anti-Inflammatory Mouthwash; TAC, tacrolimus; PDT, photodynamic therapy; CYC, cyclosporin; FLU, fluocinonide; MES; mesalazine; TRI, triamcinolone.

### Quality of the included studies

**Figure 2:**
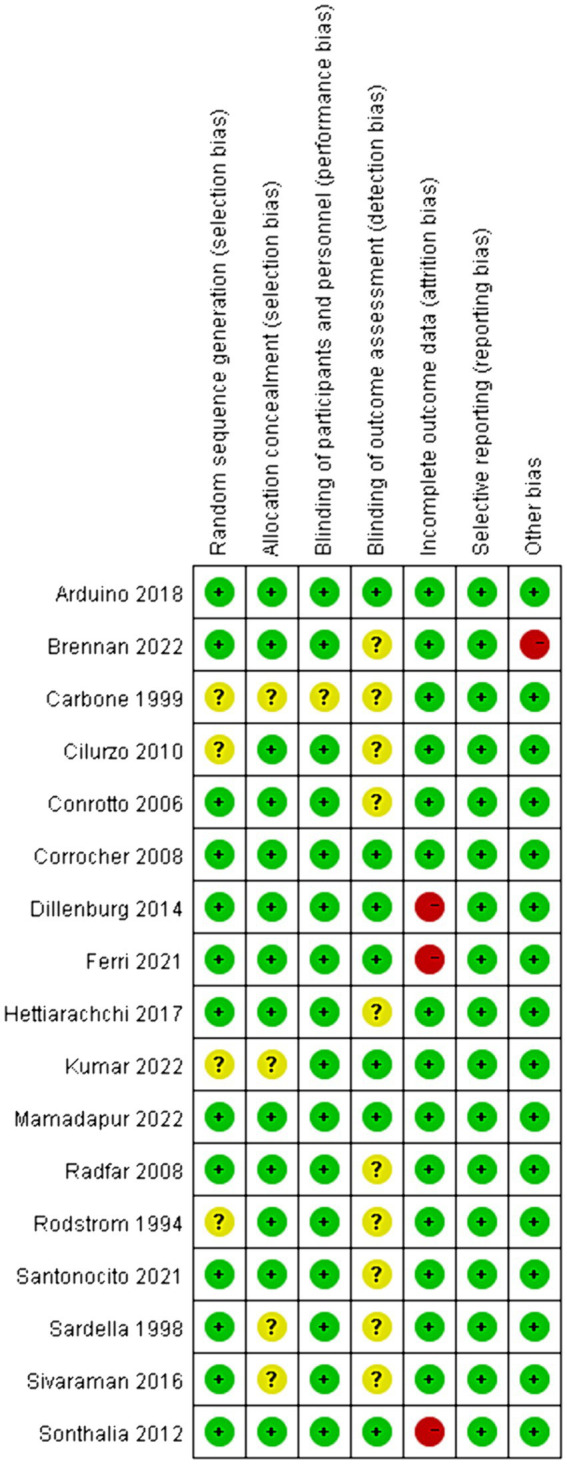
Risk-of-bias summary. Green, low risk; red, high risk; yellow, unclear risk.

The risk of bias assessments in the included studies was presented in Figure 2. Concerning random sequence generation, 4 out of the 17 studies failed to furnish a clear methodological description, resulting in an “unclear” categorization in their risk of bias assessment (20, 32, 40, 45). A similar ambiguity was observed in the domain of allocation concealment (20, 32, 37, 44). Additionally, in 16 trials, both participants and personnel adopted blinding procedures, leading us to deduce that these trials exhibited a low risk of bias. However, one trial remained ambiguous in this respect (20). In terms of outcome assessment blinding, we observed that 59% of the authors did not specify whether medical staff remained blind to the type of intervention when evaluating patient conditions (20, 31, 34, 36, 37, 40, 42–45). Yet, in 7 trials, we identified a low risk of bias due to their blinding in outcome evaluations. With regard to incomplete outcome data, the majority of studies were evaluated as low risk, but three studies were rated as high risk owing to an excessive number of dropouts (33, 38, 39). Notably, the risk of bias related to selective reporting was generally low across all reviewed studies. Upon considering other biases, we noted that one study, funded by a pharmaceutical company, was assessed as high risk (31). In summary, 4 trials were evaluated with a high risk of bias (31, 33, 38, 39), 3 with a low risk (30, 35, 41), and 10 remained unclear (20, 31, 34, 36, 37, 40, 42–45).

### Clinical score

**Figure 3:**
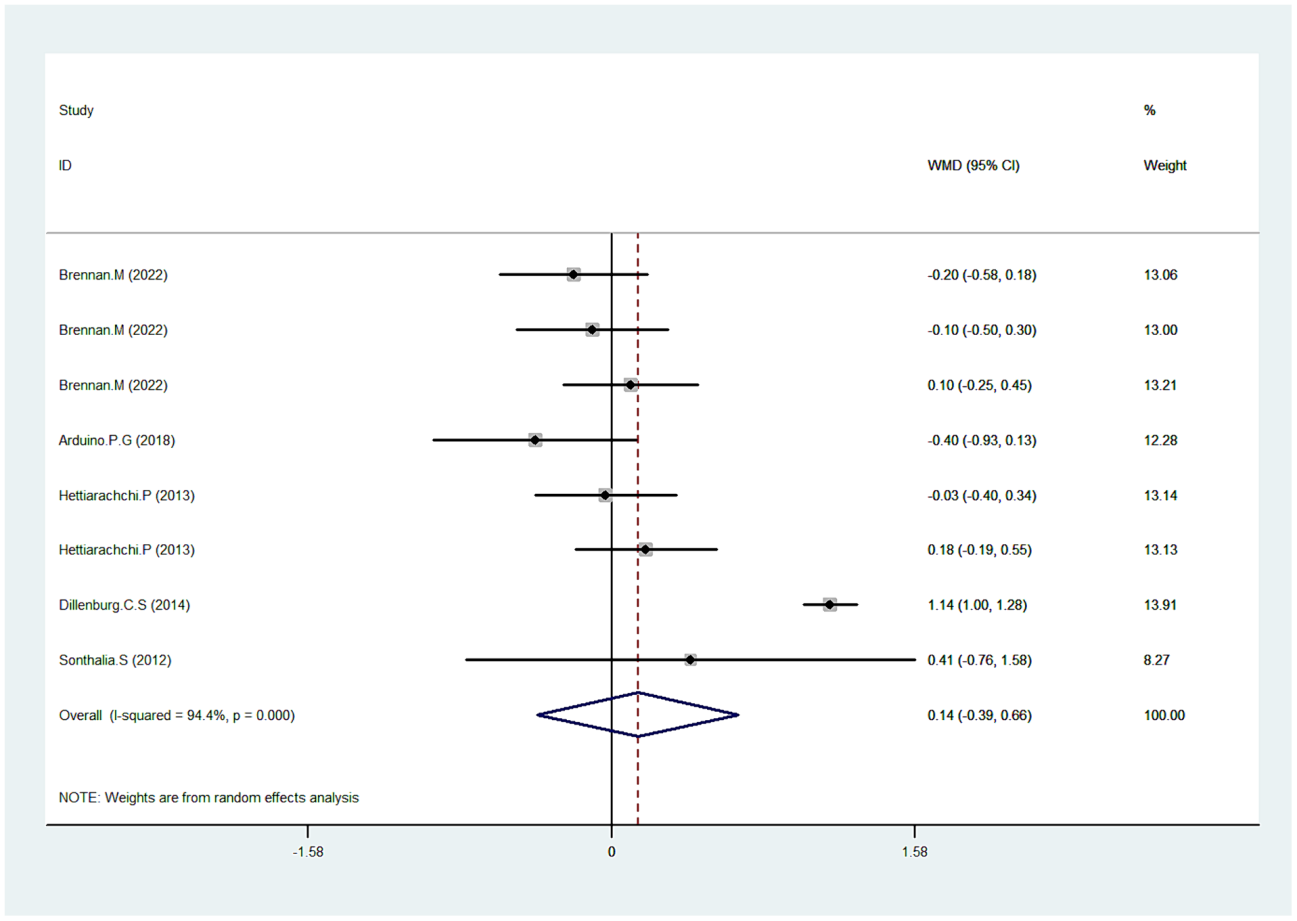
Forest plot showing the effect of CLO on the clinical score.

Five studies reported data pertaining to clinical score (31, 35, 36, 38, 39). As depicted in Figure 3, Pooled data demonstrated that CLO exhibited no significant improvement in the clinical score (WMD = 0.1495% CI: −0.39, 0.66; *p* = 0.609). However, a significant test for heterogeneity was observed (I^2^ = 94.4%, *P* < 0.001). To elucidate this inconsistency, we conducted a sensitivity analysis. Results suggested that after excluding the trial identified as an outlier (38), the heterogeneity was resolved (I^2^ = 0.0%), with the overall estimate essentially unchanged (WMD = −0.03, 95% CI: −0.19, 0.13; *p* = 0.710). This indicated that the outlier trial was the primary contributor to the observed heterogeneity.

### Clinical resolution

**Figure 4:**
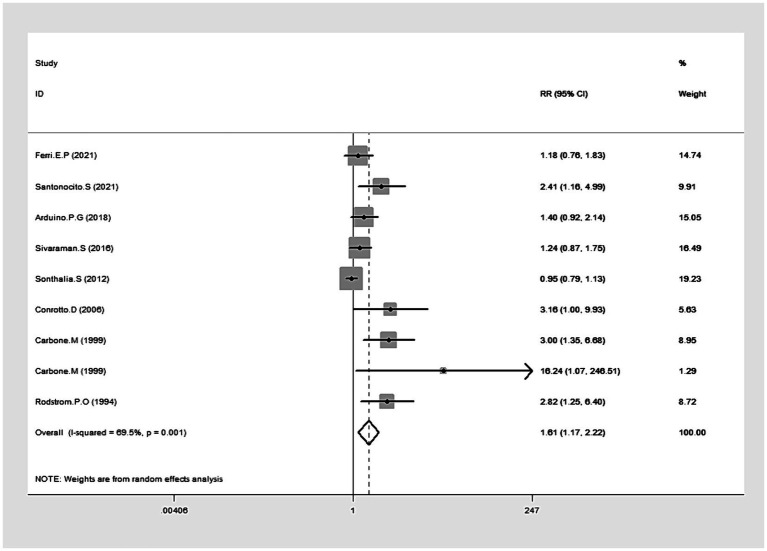
Forest plot showing the effect of CLO on the clinical resolution.

Data from eight studies provided insights into clinical resolution (20, 33–35, 37, 39, 43, 45). The collected data indicated that CLO significantly bolstered clinical resolution (RR = 1.61, 95% CI: 1.17, 2.22; *p* = 0.003), as illustrated in Figure 4. Notably, there was significant heterogeneity among these studies (I^2^ = 69.5%, *p* = 0.001). The robustness of our results was confirmed by a sensitivity analysis performed after excluding the outlier study (39): no single study substantially dominated the results (RR = 1.53, 95% CI: 1.26, 1.87; *P* < 0.001), and the heterogeneity becomes insignificant (I^2^ = 49.1%). Subgroup analyses revealed superior clinical resolution with both 0.05 and 0.025% concentrations of CLO compared to alternative treatments like CYC, FLU, TRI, and AIM. Of those, the therapeutic effects of CLO observed at 9 weeks and 26 weeks were more effective than those with shorter treatment durations (Table 2).

**Table 2:** Based on intervention treatment, control treatment and treatment duration of subgroup analysis.

Variables	Clinical score		Clinical resolution		Total lesion size	
	Overall estimate (95% CI)	*p*-value	Overall estimate (95% CI)	*p*-value	Overall estimate (95% CI)	*p*-value
**Intervention treatment**						
20 ug/patch CLO	−0.20 (−0.58, 0.18)	0.308	NA	NA	−2.37 (−7.43, 2.69)	0.359
5 ug/patch CLO	−0.10 (−0.50, 0.30)	0.621	NA	NA	−1.08 (−5.73, 3.57)	0.649
1 ug/patch CLO	0.10 (−0.25, 0.45)	0.577	NA	NA	−1.99 (−6.93, 2.95)	0.430
0.05% CLO	0.27 (−0.45, 0.98)	0.462	1.53 (1.12, 2.11)	0.009	−0.55 (−1.00, −0.09)	0.018
0.025% CLO	NA	NA	3.16 (1.00, 9.93)	0.049	NA	NA
**Control treatment**						
PBO	−0.10 (−0.31, 0.10)	0.306	3.25 (0.33, 31.81)	0.311	−1.77 (−4.59, 1.04)	0.217
TAC	0.09 (−0.16, 0.34)	0.487	1.04 (0.81, 1.33)	0.777	−0.02 (−1.61, 1.57)	0.982
CYC	NA	NA	3.16 (1.00, 9.93)	0.049	NA	NA
FLU	NA	NA	3.00 (1.35, 6.68)	0.007	NA	NA
TRI	NA	NA	2.82 (1.25, 6.40)	0.013	NA	NA
MES	NA	NA	NA	NA	NA	NA
AIM	NA	NA	2.41 (1.16, 4.99)	0.018	NA	NA
Coconut	NA	NA	NA	NA	−0.19 (−1.79, 1.41)	0.816
N. sativa	NA	NA	NA	NA	−0.63 (−1.13, −0.14)	0.012
PDT	1.14 (1.00, 1.28)	<0.001	1.18 (0.76, 1.83)	0.456	NA	NA
**Treatment duration**						
4 weeks	−0.06 (−0.27, 0.16)	0.617	1.18 (0.76, 1.83)	0.456	−0.58 (−1.97, 0.82)	0.416
5 weeks	0.07 (−0.19, 0.33)	0.576	NA	NA	NA	NA
6 weeks	NA	NA	1.24 (0.87, 1.75)	0.231	−0.58 (−1.03, −0.13)	0.016
8 weeks	−0.40 (−0.93, 0.13)	0.139	1.75 (0.86, 3.58)	0.123	NA	NA
9 weeks	NA	NA	2.82 (1.25, 6.40)	0.013	NA	NA
12 weeks	1.02 (0.48, 1.55)	<0.001	1.41 (0.57, 3.48)	0.457	NA	NA
17 weeks	NA	NA	NA	NA	NA	NA
26 weeks	NA	NA	4.15(1.13, 15.25)	0.032	NA	NA

**Variables**	**Pain score**		**Symptoms improvement**		**Adverse effects**	
	**Overall estimate (95% CI)**	**P-value**	**Overall estimate (95% CI)**	**P-value**	**Overall estimate (95% CI)**	**P-value**
**Intervention treatment**						
20 ug/patch CLO	−0.10 (−1.24, 1.04)	0.864	2.13 (1.29, 3.53)	0.003	0.94 (0.56, 1.58)	0.814
5 ug/patch CLO	0.10 (−1.00, 1.20)	0.859	NA	NA	0.97 (0.58, 1.62)	0.915
1 ug/patch CLO	0.00 (−1.07, 1.07)	1.000	NA	NA	0.88 (0.53, 1.47)	0.619
123 ug/semisolid CLO	NA	NA	3.67 (1.25, 10.71)	0.018	13.00 (0.79, 213.09)	0.072
24 ug/tablet CLO	NA	NA	4.33 (1.52, 12.34)	0.006	NA	NA
0.05% CLO	0.22 (−0.52, 0.96)	0.560	1.36 (0.77, 2.41)	0.292	2.42 (0.61, 9.56)	0.208
0.025% CLO	NA	NA	2.11 (0.76, 5.86)	0.154	6.32 (0.84, 47.69)	0.074
**Control treatment**						
PBO	−0.24 (−0.92, 0.43)	0.481	2.83 (1.81, 4.43)	<0.001	0.97 (0.72, 1.30)	0.830
TAC	0.29 (0.05, 0.53)	0.020	0.33 (0.04, 3.06)	0.332	0.43 (0.01, 17.40)	0.657
CYC	NA	NA	2.11 (0.76, 5.86)	0.154	6.32 (0.84, 47.69)	0.074
FLU	NA	NA	2.67 (1.32, 5.39)	0.006	7.00 (0.38, 127.32)	0.189
TRI	NA	NA	1.50 (0.95, 2.36)	0.081	NA	NA
MES	NA	NA	1.05 (0.52, 2.12)	0.897	NA	NA
AIM	NA	NA	2.22 (0.22, 22.49)	0.499	13.00 (0.78, 216.39)	0.074
Coconut	0.97 (0.04, 1.90)	0.040	NA	NA	NA	NA
N. sativa	−0.34 (−0.76, 0.08)	0.113	NA	NA	NA	NA
PDT	0.78 (−2.05, 3.61)	0.591	NA	NA	11.00 (0.65, 187.17)	0.097
**Treatment duration**						
4 weeks	0.31 (−0.21, 0.84)	0.243	2.20 (1.23, 3.95)	0.008	0.92 (0.57, 1.49)	0.738
5 weeks	0.28 (0.04, 0.53)	0.024	NA	NA	NA	NA
6 weeks	−0.29 (−0.70, 0.12)	0.166	0.07 (0.00, 1.08)	0.056	NA	NA
8 weeks	−1.80 (−3.53, −0.07)	0.042	2.11 (0.76, 5.86)	0.154	2.82 (1.10, 7.24)	0.031
9 weeks	NA	NA	1.50 (0.95, 2.36)	0.081	7.00 (0.38, 127.32)	0.189
12 weeks	2.02 (1.69, 2.35)	<0.001	0.77 (0.48, 1.23)	0.277	11.97 (1.63, 88.08)	0.015
17 weeks	−0.90 (−3.13, 1.32)	0.428	NA	NA	NA	NA
26 weeks	NA	NA	3.23 (1.43, 7.34)	0.005	NA	NA

NA, not available; CLO, clobetasol; PBO, placebo; N. sativa, Nigella sativa; AIM, Anti-Inflammatory Mouthwash; TAC, tacrolimus; PDT, photodynamic therapy; CYC, cyclosporin; FLU, fluocinonide; MES; mesalazine; TRI, triamcinolone.

### Total lesion size

**Figure 5:**
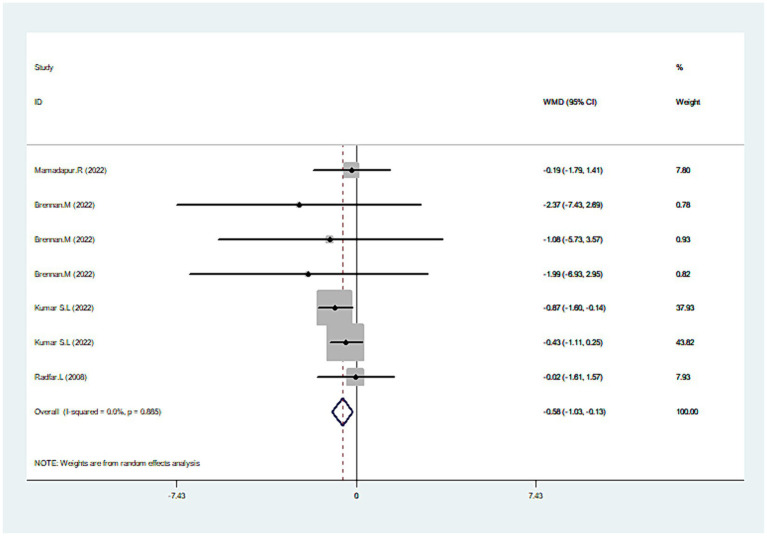
Forest plot showing the effect of CLO on the total lesion size.

Combining the results from four studies (30–32, 42), there was a significant reduction in the total lesion size with CLO treatment (WMD = −0.5895% CI: −1.03, −0.13; *p* = 0.011), as seen in Figure 5. No significant heterogeneity was revealed (I^2^ = 0.0%, *p* = 0.885), which indicated the consistency of results among these studies. An in-depth subgroup analysis was performed that the common concentration of 0.05% CLO was particularly effective in diminishing the total lesion size (Table 2).

### Pain score

**Figure 6:**
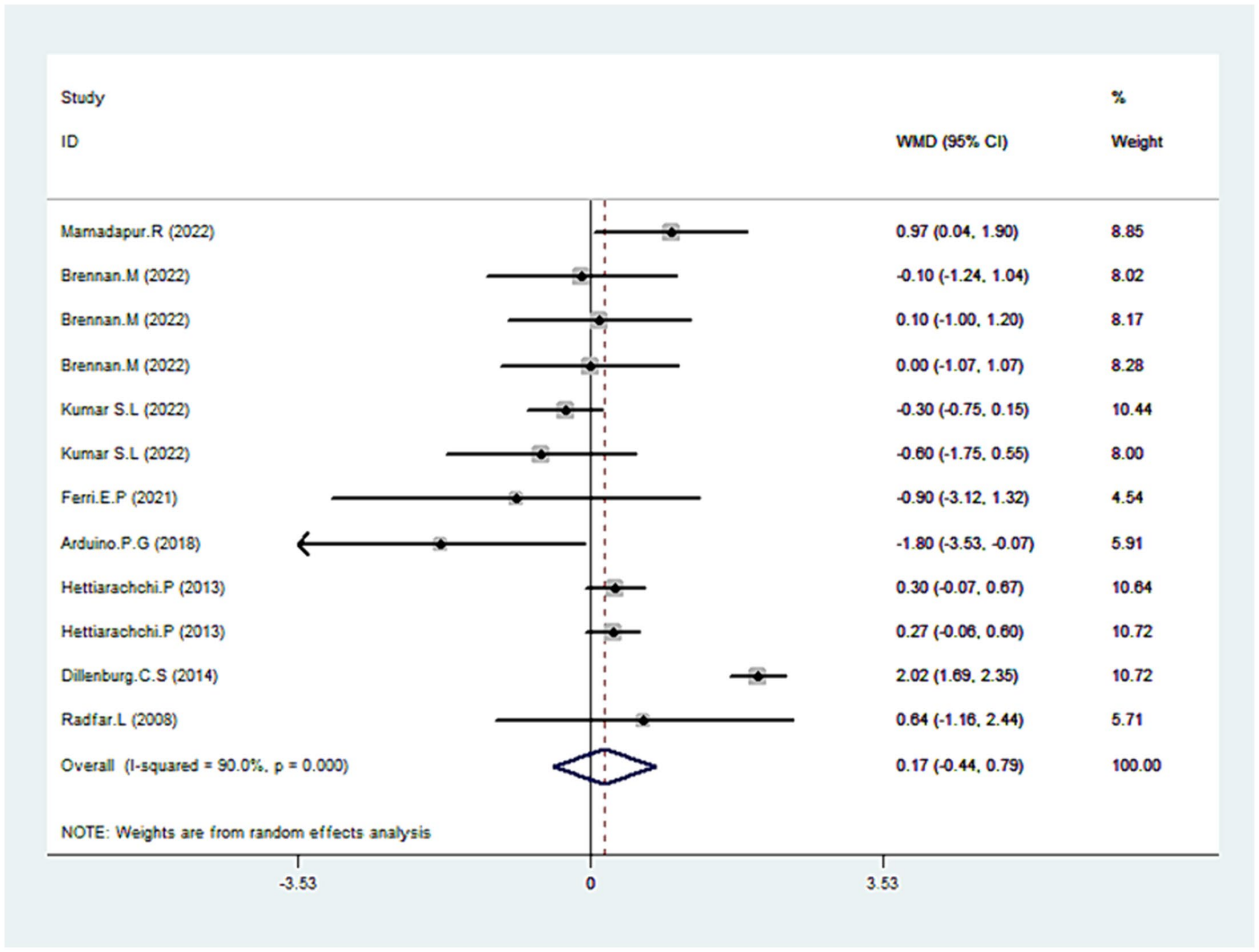
Forest plot showing the effect of CLO on the pain score.

Eight trials reported data on pain scores (30–33, 35, 36, 38, 42). As illustrated in Figure 6, CLO therapy did not produce significant variations in pain score (WMD = 0.1795% CI: −0.44, 0.79; *p* = 0.582). However, the test for heterogeneity was significant (I^2^ = 90.0%, *p* < 0.001). Sensitivity analyses were conducted to determine the robustness of the pooled results. On exclusion of the extreme outlier (38), the heterogeneity was substantially reduced (I^2^ = 36.7%), yet the overall estimated remained statistically non-significant (WMD = 0.06, 95% CI: −0.23, 0.36; *p* = 0.672). Further subgroup analysis revealed that other treatments like TAC or Coconut demonstrated superior efficacy over CLO in pain mitigation (Table 2).

### Symptoms improvement

**Figure 7:**
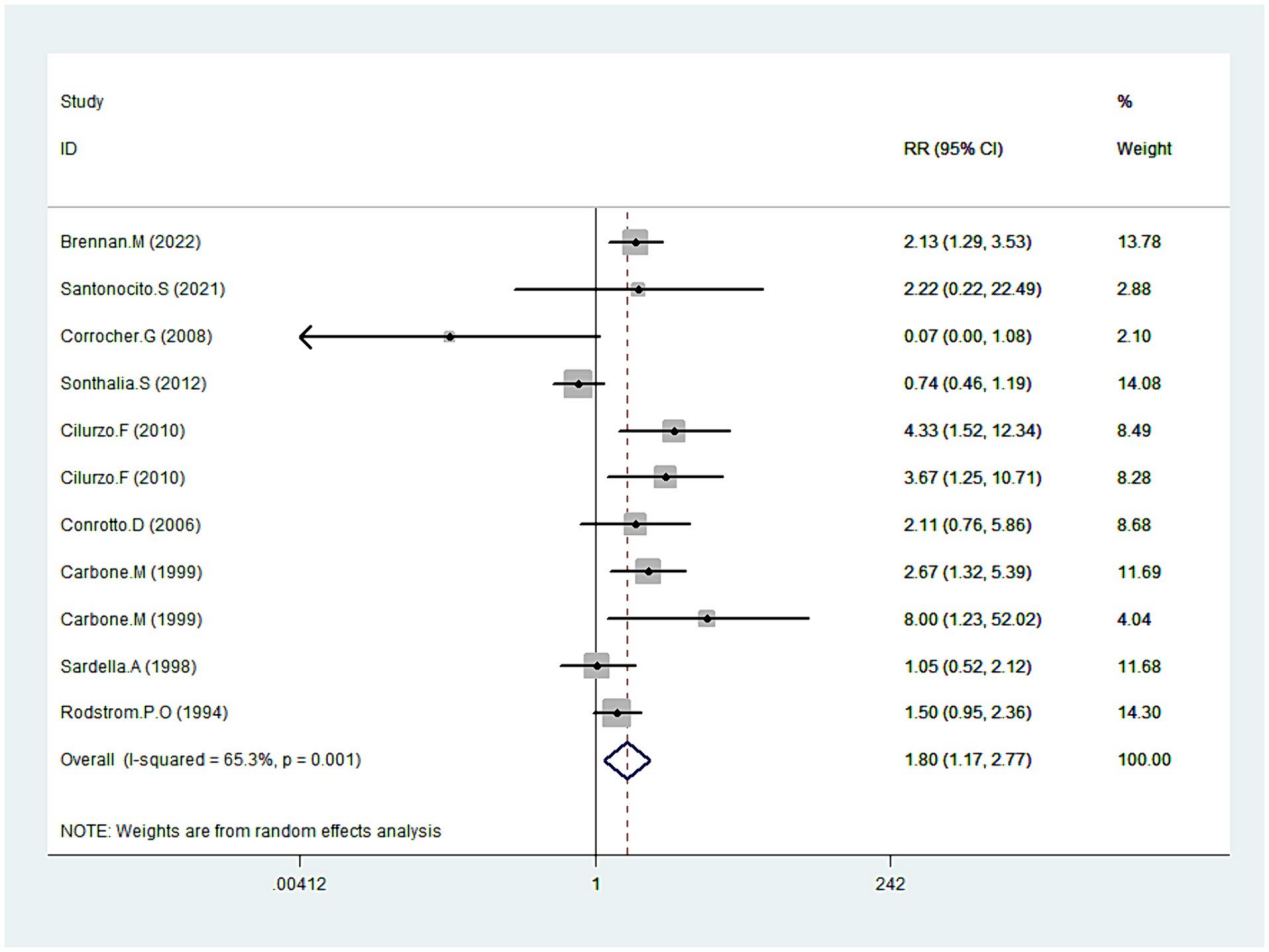
Forest plot showing the effect of CLO on the symptoms improvement.

Data on symptom improvement was offered by nine studies (20, 31, 34, 39–41, 43–45). Analysis revealed that symptoms improved notably during CLO treatment (RR = 1.8095% CI: 1.17, 2.77; *p* = 0.008), detailed in Figure 7. But still, pronounced heterogeneity was detected with this analysis (I^2^ = 65.3%, *p* = 0.001). In sensitivity analysis, heterogeneity was reduced to 46.1% by excluding the outlier trial (39), with the overall estimate essentially unchanged (RR = 2.00, 95% CI: 1.55, 2.58; *p* < 0.001). Additional subgroup analyses underscored that different dosage forms of CLO, including patch, semisolid, and tablet, demonstrated more pronounced effects on symptom improvement. Moreover, these therapeutic effects also showed significant differences when compared with PBO and FLU (Table 2).

### Relapse

Only three studies presented relapse data (20, 38, 43). The aggregated results did not indicate that CLO treatment led to a significant relapse increase (RR = 1.56, 95% CI: 0.66, 3.71; *p* = 0.314).

### Adverse effects

**Figure 8:**
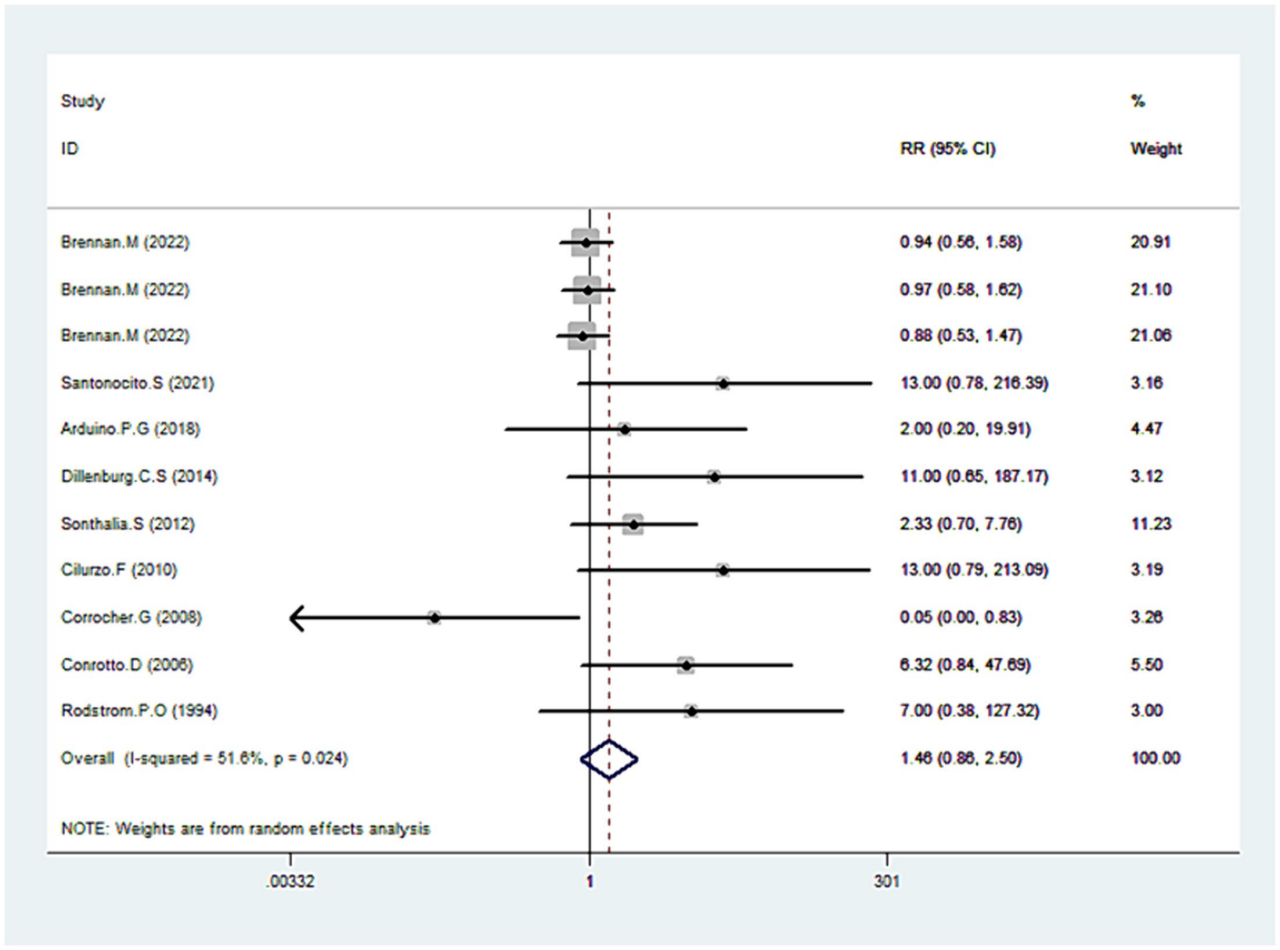
Forest plot showing the effect of CLO on the adverse effects.

Nine studies’ detailed data provided comprehensive data regarding adverse effects (31, 34, 35, 38–41, 43, 45). Our analysis revealed no statistically significant difference between CLO and other therapies in terms of adverse effects (RR = 1.46, 95% CI: 0.86, 2.50; *p* = 0.161), shown in Figure 8. However, there was notable heterogeneity among the included studies (I^2^ = 51.6%, *p* = 0.024). To address this, we excluded an outlier from the dataset, which resulted in reduced heterogeneity (I^2^ = 43.7%) without a significant change in the overall risk estimate (RR = 1.12, 95% CI: 0.85, 1.48; *p* = 0.409). Upon a detailed examination of specific adverse effects, no significant difference was observed between the CLO and other therapy groups (Table 3).

**Table 3:** Meta-analysis results of adverse effects.

Adverse events	RR (95% CI)	*p*-value
Allergy to chemicals	0.53 (0.13, 2.17)	0.380
Burning sensation	0.68 (0.30, 1.52)	0.346
Dysgeusia	5.48 (0.71, 41.97)	0.102
Gastrointestinal disorders	1.17 (0.74, 1.83)	0.500
Increase in blood sugar levels	2.57 (0.28, 23.75)	0.405
Oral candidiasis	1.97 (0.67, 5.83)	0.219
Oral fungal infection	3.31 (0.57, 19.33)	0.183
Skin rashes	1.79 (0.39, 8.14)	0.451
Salivary hypersecretion	1.37 (0.40, 4.64)	0.617

## Discussion

Our meta-analysis has evaluated the efficacy and safety of CLO in treating OLP compared to other therapeutic options. The results indicated that OLP patients who underwent CLO treatment experienced notable improvements in both lesion size and symptomatic relief, as well as in overall clinical outcomes. Previous studies have shown that CLO is particularly effective for erosive and atrophic forms of OLP, significantly reducing mucosal inflammation and stabilizing the epithelial barrier (46). Conversely, reticular OLP, characterized by milder symptoms, generally does not require potent topical corticosteroid treatment (47). Although significant heterogeneity was present in this study, potentially due to variations in treatment regimens, duration, and baseline patient conditions, the application of the sensitivity analysis successfully mitigated these potential influences, thereby enhancing the stability and reliability of the results.

In this updated search, we included eight more trials, building upon the previous network meta-analysis (NMA) (48). Unlike the previous NMA that found no clear clinical advantage of CLO over other treatments, our current meta-analysis distinctively underscores CLO’s superiority. It emerges as more effective in clinical resolution than therapies such as CYC, FLU, TRI, and AIM. This disparity can be attributed to the inclusion of newer trials and data with varied follow-up durations (48). Firstly, our study encompassed a larger number of relevant trials. Secondly, the research that was included had a longer treatment duration. Thirdly, some data that had slipped through the previous study were fortunately captured, enriching our analysis (20, 45). Another meta-analysis indicated that CLO’s role in pain management wasn’t as prominent as with PBO. This conclusion, however, mainly came from a single RCT with a small sample size, making its findings inconclusive (47). Conversely, by incorporating three additional RCTs, we found that CLO significantly outperforms placebo in alleviating symptoms (20, 31, 40). Regarding relapse rates and side effects, our findings differed from an earlier study. The prior NMA implied that CLO treatment might elevate relapse risks and adverse events (49). However, a newly included phase II RCT comprehensively evaluated side effects (31). Along with a more substantial sample size, our research offers robust evidence, suggesting a minimal link between CLO and adverse outcomes. Additionally, drawing from three newly added RCTs, ours is the inaugural meta-analysis to probe CLO’s efficiency in reducing overall lesion size (30–32).

In this study, we have observed that the clinical score of OLP patients remained consistent after undergoing CLO treatment, aligning with existing literature (31). Despite this, our subgroup analysis over a 12-week period revealed that the efficacy of PDT significantly surpassed that of CLO (*P* < 0.001). Supporting this, Dillenburg et al. (38) found that, over a prolonged treatment duration, the group subjected to PDT demonstrated a more pronounced decrease in clinical score compared to the CLO group (*P* < 0.001). However, it’s important to note two significant concerns. Firstly, the safety of PDT remains under scrutiny. There are studies that suggest that PDT might induce genomic instability, potentially heightening carcinogenesis risks (50). Secondly, the financial strain of PDT treatment is significant. Considering the initial costs of equipment and specialist training, PDT proves to be more expensive than CLO 39). Therefore, balancing economic feasibility with safety considerations positions CLO as a more appealing choice for OLP therapy.

Then, we turned our focus to assessing the ability of CLO to induce clinical resolution in OLP. Across the studies included in our meta-analysis, the mean treatment duration was 11 weeks, with a range of 4–26 weeks. In our meta-analysis, the concentration of CLO primarily used was 0.05%, which is consistent with its common application in treating OLP. A few studies also explored the efficacy of a lower concentration of 0.025%, contributing to the comprehensive evaluation of CLO’s therapeutic potential in our analysis. Although the effectiveness is somewhat modest in clinical score, two CLO concentrations (0.05% and 0.025%) both significantly outperformed other treatments, such as CYC, FLU, TRI, and AIM, in achieving clinical resolution. However, this promising finding does not come without its nuances; some studies painted a different picture. Arduino et al. (35) recognized CLO’s advantageous role in pain alleviation, yet they did not note a similar enhancement in clinical resolution. In contrast, Carbone et al. observed favorable results with CLO in both pain relief and clinical resolution (20). This disparity may rest in methodological nuances: Arduino’s study had a smaller sample size and shorter follow-up interval, leading to missing the optimal therapeutic effects of CLO. Indeed, our subgroup analysis definitively confirmed that a longer follow-up duration is more likely to highlight the benefits of CLO therapy. Hence, for patients seeking long-term treatment, CLO is a preferable choice.

In this research, we first evaluated the alteration in total lesion size in patients with OLP after CLO therapy. Echoing the sentiments of previous RCTs (32), CLO effectively shrank the total lesion size. Yet, Brennan et al. (31) contended that CLO did not offer a clear advantage over PBO in terms of lesion size reduction. This difference in findings might stem from Brennan’s decision to use the patch form of CLO, instead of the more commonly used ointment form. This choice could influence CLO’s efficacy. Our subgroup analysis supports this hypothesis, suggesting that for patients with larger lesion areas, the traditional ointment form of CLO is more advantageous.

Pain management emerges as a paramount goal in the treatment of OLP. However, clinical trials have shown that CLO alone has not yielded significant improvements in pain scores. Strikingly, a study by Brennan et al. (31) reported that a 20 μg/patch of CLO outperformed PBO in pain score. The efficacy of patch-based CLO in pain control is likely attributed to its precision, allowing for fine control of dosage and duration to ensure prolonged contact with the oral mucosa. Additionally, Ferri et al. (33) emphasized the sustained pain-relieving efficacy of CLO with extended follow-up periods. Consequently, CLO emerges as a favorable therapeutic option for long-term pain management in OLP. Furthermore, subgroup analysis, based on the choice of control treatment, revealed that both Coconut and TAC exhibited efficacy in alleviating pain. However, the TAC protocol involved intermittent long-term treatments, heightening the risk of adverse effects, notably its potential carcinogenicity (36). Conversely, Coconut faces a significant limitation due to the scarcity of evidence from RCTs, with only one referenced trial (30). As a result, it is a choice that strikes a balance between efficacy and safety to prescribe CLO to patients, given its enhanced safety profile and validation through multiple RCTs.

Regarding symptoms improvement, CLO has demonstrated significant efficacy, as corroborated by multiple studies (20, 31). Conversely, Sardella et al. (44) highlighted that MES outperformed CLO in symptoms improvement. This difference might arise from the adhesive base in MES, enhancing its adhesion to damaged tissues. Such an adhesive property is crucial in shielding the oral mucosa and reducing pain. Our subgroup analysis, based on the intervention treatments, further emphasized the significance of adhesive duration for symptom relief. The tablet, semisolid, and patch forms of CLO were more effective than the commonly used ointment form, primarily due to their extended adhesive durations. Interestingly, the non-traditional CLO formulations provided pain relief in the early phases of OLP treatment (at 4 and 6 weeks), whereas the 0.05% ointment CLO was more beneficial in later stages (at 26 weeks). Hence, for OLP patients struggling with overwhelming pain, an initial burst therapeutic intervention using patch CLO for swift pain management, segueing into a prolonged regimen of 0.05% ointment CLO, seems to be an effective strategy. Nonetheless, this proposed pharmacological approach needs comprehensive exploration and empirical validation before widespread clinical or research application.

Our study offers robust evidence that warrants further investigation into the long-term stability and safety of CLO treatment for OLP patients. Three other RCTs noted no significant difference in patient-reported relapses between CLO and alternative treatments. Yet, Conrotto et al. (43) recommended caution with the 0.025% CLO due to its higher relapse rate, which might be attributed to its reduced stability at milder concentrations compared to more concentrated formulations. In terms of side effects, our research found no major differences between CLO and other treatments. Nonetheless, Einarsdottir et al. (18) highlights that topical application of CLO may induce severe adverse reactions, such as adrenal insufficiency. This finding underscores the importance of regular assessments of adrenal function in patients undergoing long-term treatment with topical clobetasol. Further, some studies have indicated an increased risk of candidal infections with CLO (39, 40), but this link appears inconclusive, mainly due to limited sample sizes. A subsequent, larger RCT also endorsed CLO, asserting it did not result in a heightened risk of side effects (31). However, considering the significance of minimizing side effects for patient adherence, we delved deeper into the manifestation of these effects across several RCTs. Predominant adverse effects included gastrointestinal symptoms, localized burning sensations, and oral candidiasis. In most cases, these symptoms were mild and did not obstruct the treatment (34, 38). Only one instance of medication discontinuation due to abdominal pain was deemed exceptionally rare (35). Prophylactic antifungal drugs or chlorhexidine can effectively combat oral candidiasis, with most patients reacting favorably to such combination therapies (39). Few side effects, like excessive salivation and skin rashes, could be traced back to chlorhexidine (45), emphasizing the preference for antifungals in treating candidiasis. Lastly, albeit rare, CLO might induce hypersensitivity reactions, which are reversible upon discontinuation (34). To conclude, clinicians should prioritize antifungal drugs to preemptively tackle oral candidiasis, advise patients against accidental ingestion during medication use to prevent gastrointestinal issues, and aim to reduce CLO’s contact duration with oral mucosa, minimizing potential side effects.

Our meta-analysis is not without limitations. Firstly, we confined our search to RCTs, excluding other types of studies. Coupled with most of which had a relatively small sample size, this might lead to an overestimation of the therapeutic effect. Secondly, our analysis aggregated various CLO data, including different intervention treatments, control treatments and treatment duration. Furthermore, the clinical manifestations, quantity, and ages of OLP patients in the studies varied, introducing potential heterogeneity and bias into our results. Thirdly, our study did not conduct a cost comparison between CLO and alternative therapies. Fourthly, even though specific data for subgroup analyses based on lesion severity were not provided in the trials, the size or severity of the lesions might exert a potential influence on the outcomes. Lastly, the current research on OLP generally lacks randomized well-controlled blinded Good Clinical Practice (GCP) studies, and there is an inconsistency in the use of relevant, well-developed outcome assessments across studies. This limitation hinders our ability to conduct a higher quality meta-analysis.

In conclusion, our comprehensive meta-analysis affirms that CLO treatment offers significant relief from pain and clinical symptoms in patients, underscoring its therapeutic potential. On the safety front−a paramount aspect in medical research—the post-CLO treatment data indicates that both the relapse rate and incidence of adverse effects are within acceptable bounds. While there have been accounts of occasional adverse effects, most are mild, and the presence of effective countermeasures further bolsters the treatment’s safety profile. Despite the insights gained, this study has its limitations. To solidify our conclusions and delve deeper into the long-term efficacy and safety of CLO, more extensive multicenter, prospective RCTs are imperative.

## Data availability statement

The original contributions presented in the study are included in the article/supplementary material, further inquiries can be directed to the corresponding author.

## Author contributions

TZ: Conceptualization, Data curation, Investigation, Methodology, Writing – original draft, Writing – review & editing. CL: Data curation, Writing – original draft. YW: Data curation, Methodology, Writing – original draft. RZ: Conceptualization, Formal analysis, Writing – review & editing. DW: Conceptualization, Supervision, Writing – review & editing. JT: Conceptualization, Supervision, Writing – review & editing. KZ: Conceptualization, Data curation, Formal analysis, Supervision, Writing – original draft, Writing – review & editing.
